# Developing a Cancer Digital Twin: Supervised Metastases Detection From Consecutive Structured Radiology Reports

**DOI:** 10.3389/frai.2022.826402

**Published:** 2022-03-02

**Authors:** Karen E. Batch, Jianwei Yue, Alex Darcovich, Kaelan Lupton, Corinne C. Liu, David P. Woodlock, Mohammad Ali K. El Amine, Pamela I. Causa-Andrieu, Lior Gazit, Gary H. Nguyen, Farhana Zulkernine, Richard K. G. Do, Amber L. Simpson

**Affiliations:** ^1^School of Computing, Queen's University, Kingston, ON, Canada; ^2^Department of Radiology, Memorial Sloan Kettering Cancer Center, New York, NY, United States; ^3^Department of Graduate Medical Education, Memorial Sloan Kettering Cancer Center, New York, NY, United States; ^4^Department of Strategy and Innovation, Memorial Sloan Kettering Cancer Center, New York, NY, United States; ^5^Department of Biomedical and Molecular Sciences, Queen's University, Kingston, ON, Canada

**Keywords:** digital twins, cancer, metastases, machine learning, radiology, natural language processing (NLP), convolutional neural network (CNN), recurrent neural network (RNN)

## Abstract

The development of digital cancer twins relies on the capture of high-resolution representations of individual cancer patients throughout the course of their treatment. Our research aims to improve the detection of metastatic disease over time from structured radiology reports by exposing prediction models to historical information. We demonstrate that Natural language processing (NLP) can generate better weak labels for semi-supervised classification of computed tomography (CT) reports when it is exposed to consecutive reports through a patient's treatment history. Around 714,454 structured radiology reports from Memorial Sloan Kettering Cancer Center adhering to a standardized departmental structured template were used for model development with a subset of the reports included for validation. To develop the models, a subset of the reports was curated for ground-truth: 7,732 total reports in the lung metastases dataset from 867 individual patients; 2,777 reports in the liver metastases dataset from 315 patients; and 4,107 reports in the adrenal metastases dataset from 404 patients. We use NLP to extract and encode important features from the structured text reports, which are then used to develop, train, and validate models. Three models—a simple convolutional neural network (CNN), a CNN augmented with an attention layer, and a recurrent neural network (RNN)—were developed to classify the type of metastatic disease and validated against the ground truth labels. The models use features from consecutive structured text radiology reports of a patient to predict the presence of metastatic disease in the reports. A single-report model, previously developed to analyze one report instead of multiple past reports, is included and the results from all four models are compared based on accuracy, precision, recall, and F1-score. The best model is used to label all 714,454 reports to generate metastases maps. Our results suggest that NLP models can extract cancer progression patterns from multiple consecutive reports and predict the presence of metastatic disease in multiple organs with higher performance when compared with a single-report-based prediction. It demonstrates a promising automated approach to label large numbers of radiology reports without involving human experts in a time- and cost-effective manner and enables tracking of cancer progression over time.

## Introduction

Healthcare is increasingly tailoring treatments to the needs of individual patients, an approach known as personalized medicine. To achieve this, the engineering concept of Digital Twins is proposed to develop virtual patients that can be computationally treated to find optimal treatment strategies (Björnsson et al., 2020). These models are *in silico* high-resolution representations of an individual based on available molecular, physiological, and other data, which has the potential for vast improvements in patient care (Björnsson et al., 2020; Croatti et al., 2020). Personalized medicine stems from the assumption that refined mathematical models of patients will result in more precise and effective medical interventions (Bruynseels et al., 2018). This approach uses fine-grained information on individuals to identify deviations from the individual's normal to develop or select treatment focusing on a patient's individual clinical characterization such as diversity of symptoms, severity, and genetic traits, as well as environmental and lifestyle factors over time (Bruynseels et al., 2018; National Institutes of Health, 2020). Previously believed to be impossible, digital models of patients are becoming a reality with the wide-spread availability of molecular as well as other clinical data and substantial increase in computational power.

Much of the information contained in a medical record is in the form of free-text or semi-structured text data from clinical notes. Radiology reports in particular capture information critical to the treatment and management of cancer patients. Therefore, the development of a Cancer Digital Twin from routinely acquired radiology reports offers a unique opportunity to study cancer response and progression throughout a patient's journey. Manual extraction of data from CT reports requires substantial domain expertise and is prohibitively time-consuming to perform across all cancer patients. As a result, little is known about metastatic progression outside of cancer clinical trials, where response rates are most typically calculated. Data extraction from radiology reports by natural language processing (NLP) is now increasingly performed (Pons et al., 2016), including in large populations of patients with cancer, so the potential application to Digital Twins is attractive. To date, the application of NLP to radiology reports for the classification of metastatic disease has been limited to bone and brain metastases (Senders et al., 2019; Groot et al., 2020) or generalized cancer outcomes (Kehl et al., 2019). We previously presented an ensemble voting model to detect metastases from individual radiology reports for different organs using NLP (Do et al., 2021). This model considered only single reports for a given patient using standard term frequency-inverse document frequency (TF-IDF) techniques. The application of NLP to large-scale labeling of CT reports would facilitate the development of a Digital Twin and offer new insights into patterns of metastatic progression across cancer sites. Identification of such patterns will allow for the development of the high-resolution representation required for virtual patients to be effective when modeling a cancer patient's disease progression over time. This coupled with the generation of a large database of patterns of spread, early detection, and prediction of how an individual patient will progress will be possible.

Time is a critical aspect of medical data. *When* an event occurs, or the order of events that occurred, is as important as the events themselves. Studies have been conducted to incorporate the information contained in free-text clinical notes with temporal data points for ICU-related tasks (Caballero Barajas and Akella, 2015; Khadanga et al., 2019; Huang et al., 2020). Clinical notes have high-dimensionality and are sparsely recorded, creating a computational challenge compared with traditional structured time-series data (Huang et al., 2020) such as ICU data. There has been little investigation into using radiology reports throughout a patient's cancer treatment to improve the detection of metastatic spread in radiology reporting. Our research aims to fill this gap and develop a map of disease spread in individual patients over time.

In this paper, we extend our model presented in Do et al. (2021) to incorporate consecutive, multi-report prediction using several convolutional and recurrent neural network (RNN) approaches to improve detection accuracy. We present three NLP models that generate weak labels for semi-supervised classification of CT reports when exposed to multiple consecutive reports throughout a patient's treatment history.

## Materials and Methods

### Dataset Description

**Figure 1:**
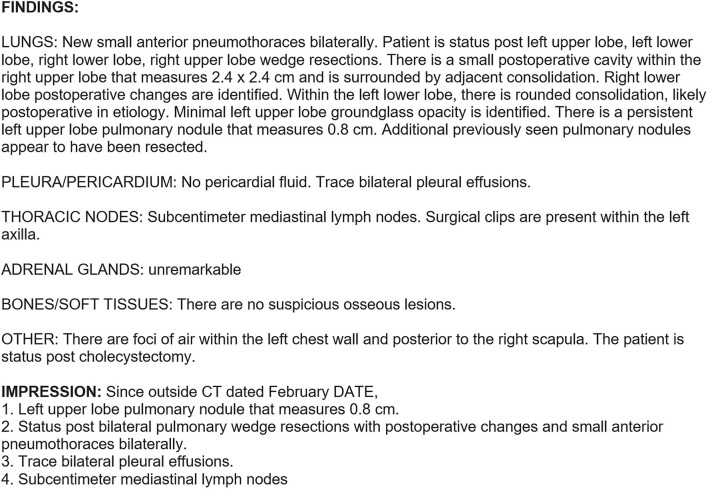
Example report of a chest CT following the template implemented in July 2009. The “Findings” section contains observations specific to each organ sites, while the “Impression” section can contain observations pertaining to any organ.

The data for this study consists of consecutive radiology reports for CT examinations of the chest, abdomen, and pelvis, performed between July 1, 2009, and March 26, 2021, at Memorial Sloan Kettering Cancer Center (MSKCC). Only reports following the departmental standardized structured template introduced in July 2009 were included; any reports which deviated from the template were omitted from analysis. The complete dataset includes 714,454 reports. Each report consists of “findings” for 13 individual organs (lungs, pleura, thoracic nodes, liver, spleen, adrenal glands, renal, abdominopelvic nodes, pelvic organs, bowel, peritoneum, bones, and soft tissues) and an overall “impression” field. The reports in this dataset are semi-structured as shown in Figure 1. In the findings section, the radiologists report observations using free text under individual headings for each organ (e.g., lung, liver). Important findings are summarized using free text in the impression section at the end of the report. Of note, non-observations are often as important as observations. This is to say that if there are “no changes” reported for a certain organ, it could mean that in a previous report metastases were identified, and they remain as they were. It could also mean that there are no lesions of interest. Standardized reporting improves clarity and consistency of clinical reports and is increasingly preferred compared to free-text reports (Renshaw et al., 2018).

Three of the 13 available organs were selected for the study: lungs, liver, and adrenal glands. The lungs and liver were selected as the most common sites of metastases while adrenal glands are one of the least common sites. Subsets of the complete dataset were annotated for ground truth by a radiologist. Each report in the ground truth set was labeled for the presence or absence of metastases. For each patient in the ground truth set, five radiologists were instructed to read all reports available before deciding the presence or absence of metastases at each time point. If after reviewing all the available reports, the radiologists were unsure about the presence or absence of metastases in a particular patient, they were instructed to skip those reports. This resulted in the following number of annotated reports: 7,732 in the lung metastases dataset from 867 individual patients; 2,777 in the liver metastases dataset from 315 patients; and 4,107 in the adrenal metastases dataset from 404 patients. Annotated reports were used to train a single-report ensemble prediction model for each organ. Once the model accuracy plateaued, the dataset was deemed to be of adequate size for that organ, resulting in differing quantities of annotated reports for each organ. Each of the three datasets (lung metastases, liver metastases, adrenal metastases) were randomly split into training (70%), testing (15%), and validation (15%) sets (see Table 1). All models were trained, tested, and validated using the same data splits to ensure accurate performance comparison at each stage.

**Table 1:** Model performance results for the baseline single-report metastases prediction model and the three novel multi-report metastases prediction models.

**Model**	**Metric**	**Training**			**Testing**			**Validation**		
		**Lung** **(*n* = 5,413)**	**Liver** **(*n* = 1,943)**	**Adrenal** **(*n* = 2,874)**	**Lung** **(*n* = 1,160)**	**Liver** **(*n* = 417)**	**Adrenal** **(*n* = 617)**	**Lung** **(*n* = 1,160)**	**Liver** **(*n* = 417)**	**Adrenal** **(*n* = 616)**
TF-IDF ensemble model (Baseline)	Accuracy	99.69% (±0.15%)	**99.95% (±0.10%)**	99.23% (±0.32%)	92.33% (±1.53%)	90.12% (±2.86%)	96.60% (±1.43%)	93.80% (±1.39%)	92.50% (±2.53%)	96.10% (±1.53%)
	Precision	0.9977 (±0.00)	**1.0000 (±0.00)**	**1.0000 (±0.00)**	0.8553 (±0.02)	0.9060 (±0.03)	**0.9444 (±0.02)**	0.9080 (±0.02)	0.8990 (±0.03)	**1.0000 (±0.00)**
	Recall	0.9833 (±0.00)	0.9983 (±0.00)	0.8932 (±0.01)	0.6733 (±0.03)	0.7794 (±0.04)	0.4595 (±0.04)	0.6860 (±0.03)	0.8310 (±0.04)	0.5000 (±0.04)
	F1-score	0.9904 (±0.00)	**0.9991 (±0.00)**	0.9436 (±0.01)	0.7535 (±0.02)	0.8379 (±0.04)	0.6182 (±0.04)	0.7815 (±0.02)	0.8637 (±0.03)	0.6667 (±0.04)
Simple CNN	Accuracy	99.93% (±5.21%)	99.85% (±7.59%)	**100% (±0.00%)**	**97.41% (±0.91%)**	**98.56% (±1.14%)**	**99.03% (±0.77%)**	96.64% (±1.04%)	98.56% (±1.14%)	99.51% (±0.55%)
	Precision	0.9956 (±0.00)	0.9950 (±0.00)	**1.0000 (±0.00)**	**0.9526 (±0.01)**	0.9851 (±0.01)	0.9429 (±0.02)	**0.9526 (±0.01)**	0.9746 (±0.02)	0.9592 (±0.02)
	Recall	**1.0000 (±0.00)**	**1.0000 (±0.00)**	**1.0000 (±0.00)**	0.8960 (±0.02)	0.9706 (±0.02)	**0.8919 (±0.02)**	0.8564 (±0.02)	0.9746 (±0.02)	**0.9792 (±0.01)**
	F1-score	0.9978 (±0.00)	0.9975 (±0.00)	**1.0000 (±0.00)**	0.9234 (±0.02)	0.9778 (±0.01)	**0.9167 (±0.02)**	0.8920 (±0.02)	0.9746 (±0.02)	0.9691 (±0.02)
Augmented CNN	Accuracy	**99.98% (±0.04%)**	99.90% (±0.14%)	99.97% (±0.06%)	**97.41% (±0.91%)**	**98.56% (±1.14%)**	98.87% (±0.83%)	96.81% (±1.01%)	**99.04% (±0.94%)**	**99.68% (±0.45%)**
	Precision	**0.9989 (±0.00)**	0.9966 (±0.00)	0.9952 (±0.00)	0.9388 (±0.01)	0.9710 (±0.02)	0.9167 (±0.02)	0.9467 (±0.01)	**0.9831 (±0.01)**	**0.9792 (±0.01)**
	Recall	**1.0000 (±0.00)**	**1.0000 (±0.00)**	**1.0000 (±0.00)**	**0.9109 (±0.02)**	**0.9853 (±0.01)**	**0.8919 (±0.02)**	0.8511 (±0.02)	**0.9831 (±0.01)**	**0.9792 (±0.01)**
	F1-score	**0.9994 (±0.00)**	0.9983 (±0.00)	0.9976 (±0.00)	**0.9246 (±0.02)**	0.9781 (±0.01)	0.9041 (±0.02)	**0.8964 (±0.02)**	**0.9831 (±0.01)**	**0.9792 (±0.01)**
Bidirectional LSTM	Accuracy	97.97% (±0.38%)	99.23% (±0.39%)	99.72% (±0.19%)	96.66% (±1.03%)	**98.56% (±1.14%)**	98.70% (±0.89%)	**97.16% (±0.96%)**	98.32% (±1.23%)	99.03% (±0.77%)
	Precision	0.9052 (±0.01)	0.9798 (±0.01)	0.9660 (±0.01)	0.8465 (±0.02)	**0.9853 (±0.01)**	0.8919 (±0.02)	0.8404 (±0.02)	0.9661 (±0.02)	0.9375 (±0.02)
	Recall	0.9366 (±0.01)	0.9873 (±0.00)	0.9803 (±0.01)	0.8976 (±0.02)	0.9781 (±0.01)	**0.8919 (±0.02)**	**0.9054 (±0.02)**	0.9702 (±0.02)	0.9375 (±0.02)
	F1-score	0.9206 (±0.01)	0.9835 (±0.01)	0.9731 (±0.01)	0.8713 (±0.02)	**0.9817 (±0.01)**	0.8919 (±0.02)	0.8717 (±0.02)	0.9682 (±0.02)	0.9375 (±0.02)

*Organ datasets are split into three subsets for training (70%), testing (15%), and validation (15%). The n values correspond to the size of the sets. The highest values for each organ in each performance metric are bolded. Values in parentheses are within the 95% confidence interval rounded to two decimal places*.

#### Data Preprocessing

The raw text data consisted of organ observations from the report, each associated with a patient. To transform the data into a format for multi-report analysis, individual reports were grouped by patient and ordered chronologically from oldest to newest. For each report *r*_*t*_ of a patient, all previous reports (*t* = *0, 1, …, n*, where *n* is the target report) were concatenated into a single document. For example, if the target report is the first report associated with the patient, the resulting document would consist only of this report. If the target report is the third report associated with the patient, the resulting document consists of the patient's first, second, and third reports concatenated together in chronological order. The radiology reports often included dates and lesion measurements. These text patterns were identified using regular expressions and replaced with the text “date” and “measurement”, respectively. This is done to shrink the size of the vocabulary as well as to capture the higher-level concept of a date or measurement being present in the text. Since the measurements themselves were not included in any analysis, it was beneficial to remove them from the vocabulary space. Target values (i.e., labels) were encoded from “Yes” and “No” values to binary values 1 and 0, respectively.

### Model Development

Three models were developed to predict the presence of metastases over time in each of the three target organs namely, a simple convolutional neural network (CNN), a CNN augmented with an attention layer (referred to as the Augmented CNN), and a bidirectional Long Short-Term Memory (Bi-LSTM) model. Convolutional neural network extract spatial features which allows for the maintenance of context when analyzing text in NLP applications. Adding the attention layer to the CNN allows for increased explainability and allows the model to learn and give higher importance to features later in the sequence. The Bi-LSTM was selected because it learns context of information and the sequence of patterns by traversing the text in two directions to create superior text embedding. For the purposes of this study, we combine multiple consecutive reports of a patient consisting of observations made by a radiologist into a single document which is used as the input to the model. To evaluate the benefit of looking at multiple consecutive reports compared to only one report, the single-report model described previously by our group (Do et al., 2021) was used as a baseline. The models are compared based on the following metrics: accuracy, precision, recall, and F_1_ measure. F_1_ measure is considered the most important metric because F_1_ is the harmonic mean of precision and recall and provides a better measure of incorrectly classified cases than accuracy. In the cases of identifying potential metastases, the cost of missing positive cases (false negatives) is much greater than the cost of false positives, which is reflected in the F_1_ score. F_1_ also mitigates the effect of imbalanced class distribution, which can be masked behind accuracy scores.

#### Baseline Model

We previously presented an ensemble voting model to detect metastases from individual radiology reports for different organs using NLP (Do et al., 2021). This model is used as the baseline for performance evaluation of the multi-report prediction models presented in the current paper. Briefly, this baseline model processes the raw text data using a TF-IDF method. The processed data are passed through an ensemble voting model built with a logistic regression (LR) model, a support vector machine (SVM), a random forest (RF) model, and an extreme gradient boosting (XGBoost) model. The specifications for each model are given in the following paragraph. Ensemble models use a “voting” strategy to select the best prediction based on predictions made by multiple underlying statistical models. Voting can be done using either a hard vote counter or a soft vote counter. In hard voting, the final classification is made based on a strict count of the predictions made by the underlying models, while soft voting gives higher importance to certain models. In soft voting, the models in the ensemble are ranked using a simple weighting algorithm to determine the relative importance given to each model's predictions. The algorithm compares the accuracy, precision, and recall metrics of all models on the training set to assign the weights. These values are used such that the best-performing classification model's prediction is given the highest importance when tallying the votes. In addition to this ranked weighting, the confidence values for each model's prediction are leveraged in making the final prediction. Our model uses soft voting. Importance calculations were done for each organ to better optimize model performance by location. This means that the weights assigned to the individual models for predicting liver metastases may be different from those for predicting lung metastases.

The LR model is configured with a regularization strength of 15.0, it uses balanced class weighting, which automatically adjusts the weights inversely proportional to class frequencies in the input data. It uses the Newton-CG optimization algorithm solver to handle multinomial loss in the multiclass prediction problem. The SVM uses a linear kernel and all other default parameters. The RF model is built to have 2,000 trees with bootstrapping and the maximum number of features used when building a tree is set to the square root of the number of features seen during *fit*. The XGBoost model uses default configuration.

#### Simple Convolutional Neural Network

Text data from the radiology reports must be converted into a numeric vector representation to be used as inputs to machine learning models. Recent studies (Zuccon et al., 2015; Zhao and Mao, 2018; Verma et al., 2021) have shown different text encoding approaches having different complexities and ability to represent contextual information. One of the popular approaches is called word embedding, which includes word context and transforms each word to a numeric vector capturing semantic information (Ghannay et al., 2016). The transformation allows different words having similar meanings to have vector representations that are close together in the embedding space. For the convolutional neural network models, the text data is transformed using the Tokenizer from TensorFlow (Abada et al., 2015). Tokenizer creates a vocabulary of all the unique terms in the training corpus and allows for vectorization of the text corpus by turning each document into a sequence of integers, where each integer is the index of a token in a dictionary. All punctuation is removed from the text when it is processed through Tokenizer. When any text is processed by the Tokenizer, only the known words are processed while the unknown words are ignored. This processed data is then fed as input to the convolutional layers of the model.

The idea behind convolutions in computer vision is to learn filters that transform adjacent pixels into single values. A CNN for NLP learns which combinations of adjacent words are associated with a given concept, meaning they can augment the existing techniques by leveraging the representation of language to learn which phrases in clinical text are relevant for a given medical condition. In a CNN, a text is first represented as a sequence of word embeddings in which each word is projected into a distributed representation. Words that occur in similar concepts are trained to have similar embeddings, meaning misspellings, synonyms, and abbreviations of an original word learn similar embeddings, leading to similar results. Therefore, a database of synonyms and common misspellings is therefore not required.

Embedded text is the input to the convolutional layer. Convolutions detect a signal from a combination of adjacent inputs, and each convolution operation applies a filter of trained parameters to an input-window of specific width. A filter is applied to every possible word window in the input to produce a feature map. The feature map is then reduced using a pooling operation. It is possible to combine multiple convolutions per length and of different lengths to evaluate phrases from 1 to 5 words long, for example. A final fully connected feed forward layer helps compute the probability of whether the text refers to a patient with a certain disease condition.

**Figure 2:**
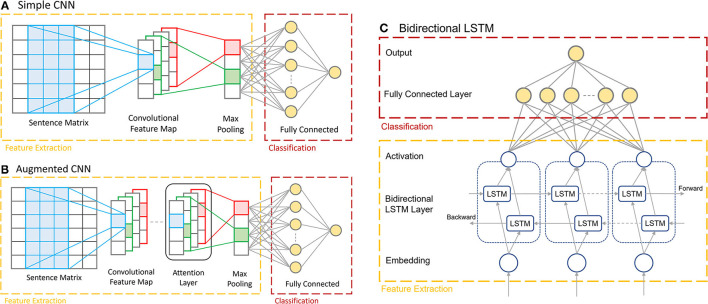
The architectures of the three multi-report prediction models. **(A)** The Simple CNN architecture consisting of the embedding layer, 1D convolutional layer, max pooling layer, and dense layers. **(B)** The Augmented CNN, consisting of the same architecture as the Simple CNN with an added Attention Layer before the max pooling layer. **(C)** The Bi-LSTM, with the two LSTM layers processing inputs in opposite directions.

The CNN model (Figure 2A) is built using Keras (Chollet, 2015), which consists of an embedding layer with an embedding dimension of 50, a 1D convolutional layer with a filter size of 64, a kernel size of 3 and using ReLU activation, followed by a global max pooling layer, and finally two fully connected dense layers containing 10 nodes and 1 node, respectively. The final single output node generates a binary decision of whether the input corresponds to the presence of a certain disease condition or not. The penultimate layer uses ReLU activation, and the ultimate layer uses sigmoid activation to make the final prediction. The model is optimized using the ADAM optimizer and the binary cross-entropy loss function.

#### Augmented CNN

The Augmented CNN (Figure 2B) consists of the same architecture as the Simple CNN model as describe above with one added layer: the Keras Sequential Self Attention layer (SeqSelfAttention). This layer implements an attention mechanism when processing sequential data to learn important text embedding and attend to that information (increase weight values) when extracting data features. It is added after the convolutional layer in the model, and its output is fed as the input to the global max pooling layer. The attention layer is configured to use multiplicative attention, an attention width of 1, and uses sigmoid attention activation. The remaining layers of the model are the same as in the Simple CNN.

#### Bi-Directional LSTM

Long Short-Term Memory (LSTM) (Hochreiter and Schmidhuber, 1997) networks are a powerful type of RNN. One of the main limitations of the basic RNNs is that they lose critical information when dealing with long sequences. Long Short-Term Memories are explicitly designed to avoid such problems and retain information from long sequences of data to learn dependencies in data that are far apart. Thus, the model can remember and also forget certain information it has previously seen. These models consist of a cell state *c*_*t*−1_ (i.e., the memory of the network) and a hidden state *h*_*t*−1_ (used to make predictions) with three gates that allow the gradient to flow unchanged. The three gates are a forget gate, an input gate, and an output gate. The *forget gate* determines what information are going to be thrown away from the cell state. This gate is essentially a sigmoid function, taking hidden state *h*_*t*−1_ and data *x*_*t*_ as input, and outputs a number between 0 and 1 for each element in the cell state. A 0 means to completely throw away the information while a 1 means keep all the detail of that element. The *input gate* determines what new information is going to be retained in the cell state (update the cell state from *c*_*t*−1_ to $\tilde{c_{t}}$). This layer has two parts: a sigmoid layer and a tanh layer. The sigmoid layer takes hidden state *h*_*t*−1_ and data *x*_*t*_ as input and determines which values to update by assigning a number between 0 and 1 to each element computed in the tanh layer. The tanh layer transforms the data *x*_*t*_ and hidden state *h*_*t*−1_ to a number between −1 and 1. Next, the product of both layers yields the update to the cell state. The cell state is updated by multiplying the output from the forget gate elementwise, ensuring only critical information can flow down the sequence. Next, the results from the input gate are added elementwise to the cell state. This completes the cell state update, yielding *c*_*t*_. We use the freshly updated cell state *c*_*t*_ to update the hidden state. In the *output gate*, it first passes the hidden state *h*_*t*−1_ and data *x*_*t*_ through a sigmoid layer. Then, *c*_*t*_ is passed through a tanh layer and these results are multiplied together, yielding the new hidden state *h*_*t*_.

Bidirectional RNN models are two combined RNN models, one model processing data sequentially from beginning to end, while the other received input data in the opposite direction, from end to start. These models perform data analysis simultaneously and their results (predictions) are combined and passed to the dense layers.

For the Bi-LSTM (Figure 2C), a self-created dictionary is used for the word embedding. Each unique word in the reports is extracted and sorted in the order of alphabet. Each word is then assigned an index, reserving the first two indices for padding (0) and unknown (1) values, respectively. This model encodes the input documents as vectors consisting of values corresponding to the word's index in the vocabulary dictionary. This means that each input vector for this model depends on the length of the original report, which is variable. The documents are not padded initially but will be padded to the same length for each batch while they are passing through the data generator function. This data is then passed through into the two LSTM layers, processing the data in opposite directions.

The Bi-LSTM was also developed using Keras. The first layer is an embedding layer with input dimension equal to the size of the vocabulary and an output size of 64. This is followed by the Bidirectional LSTM layer provided in the Keras library, which uses the tanh activation function and sigmoid recurrent activation. The output dimension of this layer is 64. The final two layers of the model are similar to those found in both the Simple and Augmented CNN models; the penultimate layer is a fully connected dense layer with 64 nodes and ReLU activation, while the ultimate layer is a dense layer with one output node with no activation.

## Results

Metastatic disease was present in 16.6% (1,287/7,733) of the reports in the lung dataset, 30.5% (848/2,777) of the reports in the liver dataset, and in 7.1% (291/4,107) of the adrenal gland dataset. These distributions were consistent in the training, testing, and validation sets. Prediction accuracies exceeded 96% across all organs and all models during validation, with the lowest accuracy being the Simple CNN predicting the presence of lung metastases. The F_1_ scores are especially promising, showing balanced precision and recall scores in all models. The F_1_ scores demonstrate that the Augmented CNN is the most balanced model, though all models' F_1_ scores for the lung dataset were below 0.90. The F_1_ scores for lung metastases detection are consistently the lowest, though always scoring above 0.87. The performance metrics of the three models on the validation dataset are presented in Table 1. We compare these results with the performance of the baseline model outlined in section Baseline Model, which predicts the presence of metastases from single reports, in contrast to the three deep learning models, which include information from previous reports concatenated in chronological order.

The training and testing results for the baseline TF-IDF Ensemble Voting model are as follows: in training, the model scored 99.69 ± 0.001%, 0.9977, 0.9833, and 0.9904 (accuracy, precision, recall, F_1_ score) on the lung dataset, 99.95%, 1.00, 0.9983, and 0.9991 (accuracy, precision, recall, F_1_ score) on the liver dataset, and 99.23%, 1.00, 0.8932, and 0.9436 (accuracy, precision, recall, F_1_ score) on the adrenal gland dataset. In testing, the model scored 92.33%, 0.8553, 0.6733, and 0.7535 (accuracy, precision, recall, F_1_ score) on the lung dataset, 90.12%, 0.9060, 0.7794, and 0.8379 (accuracy, precision, recall, F_1_ score) on the liver dataset, and 96.60%, 0.9444, 0.4595, and 0.6182 (accuracy, precision, recall, F_1_ score) on the adrenal gland dataset. During validation, the model scored 93.80%, 0.9080, 0.6860, and 0.7815 (accuracy, precision, recall, F_1_ score) on the lung dataset, 92.50%, 0.8990, 0.8310, and 0.8637 (accuracy, precision, recall, F_1_ score) on the liver dataset, and 96.10%, 1.00, 0.5000, and 0.6667 (accuracy, precision, recall, F_1_ score) on the adrenal gland dataset. The results from the baseline model are also presented in Table 1.

The complete results for the Simple CNN on each dataset are as follows: in training, the model scored 99.93%, 0.9956, 1.00, and 0.9978 (accuracy, precision, recall, F_1_ score) on the lung dataset, 99.85%, 0.9950, 1.00, and 0.9975 (accuracy, precision, recall, F_1_ score) on the liver dataset, and 100%, 1.00, 1.00, and 1.00 (accuracy, precision, recall, F_1_ score) on the adrenal gland dataset. In testing, the model scored 97.41%, 0.9526, 0.8960, and 0.9234 (accuracy, precision, recall, F_1_ score) on the lung dataset, 98.56%, 0.9851, 0.9706, and 0.9778 (accuracy, precision, recall, F_1_ score) on the liver dataset, and 99.03%, 0.9429, 0.8919, and 0.9167 (accuracy, precision, recall, F_1_ score) on the adrenal gland dataset. During validation, the model scored 96.64%, 0.9526, 0.8564, and 0.8920 (accuracy, precision, recall, F_1_ score) on the lung dataset, 98.56%, 0.9746, 0.9746, and 0.9746 (accuracy, precision, recall, F_1_ score) on the liver dataset, and 99.51%, 0.9592, 0.9792, and 0.9691 (accuracy, precision, recall, F_1_ score) on the adrenal gland dataset. The results from the Simple CNN model are also presented in Table 1.

The complete results for the Augmented CNN on each dataset are as follows: in training, the model scored 99.98%, 0.9989, 1.00, and 0.9994 (accuracy, precision, recall, F_1_ score) on the lung dataset, 99.90%, 0.9966, 1.00, and 0.9983 (accuracy, precision, recall, F_1_ score) on the liver dataset, and 99.97%, 0.9952, 1.00, and 0.9976 (accuracy, precision, recall, F_1_ score) on the adrenal gland dataset. In testing, the model scored 97.41%, 0.9388, 0.9109, and 0.9246 (accuracy, precision, recall, F_1_ score) on the lung dataset, 98.56%, 0.9710, 0.9853, and 0.9781 (accuracy, precision, recall, F_1_ score) on the liver dataset, and 98.87%, 0.9167, 0.8919, and 0.9041 (accuracy, precision, recall, F_1_ score) on the adrenal gland dataset. During validation, the model scored 96.81%, 0.9467, 0.8511, and 0.8964 (accuracy, precision, recall, F_1_ score) on the lung dataset, 99.04%, 0.9831, 0.9831, and 0.9831 (accuracy, precision, recall, F_1_ score) on the liver dataset, and 99.68%, 0.9792, 0.9792, and 0.9792 (accuracy, precision, recall, F_1_ score) on the adrenal gland dataset. The results from the Augmented CNN model are also presented in Table 1.

The complete results for the Bi-LSTM on each dataset are as follows: in training, the model scored 97.97%, 0.9052, 0.9366, and 0.9206 (accuracy, precision, recall, F_1_ score) on the lung dataset, 99.23%, 0.9798, 0.9873, and 0.9835 (accuracy, precision, recall, F_1_ score) on the liver dataset, and 99.72%, 0.9660, 0.9803, and 0.9731 (accuracy, precision, recall, F_1_ score) on the adrenal gland dataset. In testing, the model scored 96.66%, 0.8465, 0.8976, and 0.8713 (accuracy, precision, recall, F_1_ score) on the lung dataset, 98.56%, 0.9853, 0.9781, and 0.9817 (accuracy, precision, recall, F_1_ score) on the liver dataset, and 98.70%, 0.8919, 0.8919, and 0.8919 (accuracy, precision, recall, F_1_ score) on the adrenal gland dataset. During validation, the model scored 97.16%, 0.8404, 0.9054, and 0.8717 (accuracy, precision, recall, F_1_ score) on the lung dataset, 98.32%, 0.9661, 0.9702, and 0.9682 (accuracy, precision, recall, F_1_ score) on the liver dataset, and 99.03%, 0.9375, 0.9375, and 0.9375 (accuracy, precision, recall, F_1_ score) on the adrenal gland dataset. The results from the Bi-LSTM model are also presented in Table 1.

## Discussion

We developed three novel models for detecting metastatic disease in three separate organs using NLP over multiple consecutive radiology reports. Both the CNN models and the Bi-LSTM model demonstrated high performance in accomplishing this task. Our results demonstrate the added predictive power of exposing an NLP model to historical patient information. Indeed, F_1_ score increased from 0.7815, 0.8637, and 0.6667 to 0.8964, 0.9831, and 0.9792 in the lung, liver, and adrenal gland datasets, respectively, when multiple reports were considered. Accuracy, precision, and recall all improved with the multi-report model. The best-performing model—the Augmented CNN—achieved the highest F_1_ scores at all three organ sites during validation. Through the model development process, the model performance remained consistent through training, testing, and validation. During training, the models were exposed to records with varying number of concatenated reports, meaning the models have been trained to detect metastases with varying amounts of available information so the models can be used at any point within a patient's course of treatment.

Performance on the lung dataset was lowest for all three models, with F_1_ scores of 0.8920, 0.8964, and 0.8717 achieved by the Simple CNN, the Augmented CNN, and the Bi-LSTM, respectively. This is likely because the lung can be subject to a large variety of ailments, such as infections, some of which overlap in appearance with metastatic disease because they appear to radiologists as pulmonary nodules. In analyzing the model's decision-making by extracting the most predictive terms from the vocabulary, it was identified that the presence of measurements in a report were highly indicative of the presence of a metastatic disease. This is not surprising since radiologists commonly rely on measurements to document response to treatment in their report. Use of this feature is excellent in detecting metastases in the liver and adrenal glands, however there are more types of lesions that are measured for the lung, including benign lung nodules. Predicting based on the presence of measurements in the case of lung metastases therefore, results in higher frequency of false positives. The F_1_ scores for liver and adrenal metastases predictions on the validation sets exceeded 0.9691, and the Bi-LSTM was only slightly lower at 0.9375 when predicting the presence of adrenal metastases.

There are many papers that describe the usage of NLP for text mining clinical notes, linking events described in notes to time series data (typically for prediction of mortality or length of stay) (Caballero Barajas and Akella, 2015; Khadanga et al., 2019; Huang et al., 2020). A recent study used NLP and deep learning for case-level context for classifying pathology reports has demonstrated the success of CNNs, RNNs, and attention models (Gao et al., 2020), such as those presented in our study. While presenting similar models, this previous study focused on several multi-class classification problems, while our study focuses on the binary classification of the presence or absence of metastatic disease. Both studies demonstrate the benefit of capturing case-level context from consecutive reports compared with single-report prediction, however our models demonstrate higher F1 scores overall. To our knowledge, ours is the first demonstration of multi-report detection in consecutive radiology reports. Specifically, we consider the order that metastases appear for each patient by concatenating reports but do not consider the length of time between metastatic events. Given the overall high performance of our models, factoring in the actual time may not be warranted for simple detection of labels. As we advance our methods to metastatic phenotype identification, the goal of our cancer digital twin, time will likely be an important factor. When included in a digital twin, the series of metastatic cancer labels will show how the individual's state is changing over time and construct the high-resolution representations required. We were the first to demonstrate the benefit of semi-structured narrative reports in the largest study using NLP for identifying metastatic disease (Do et al., 2021), combined with the new models proposed in the current study, we are unlocking the potential of using cancer digital twins for anticipating cancer response and progression.

**Figure 3:**
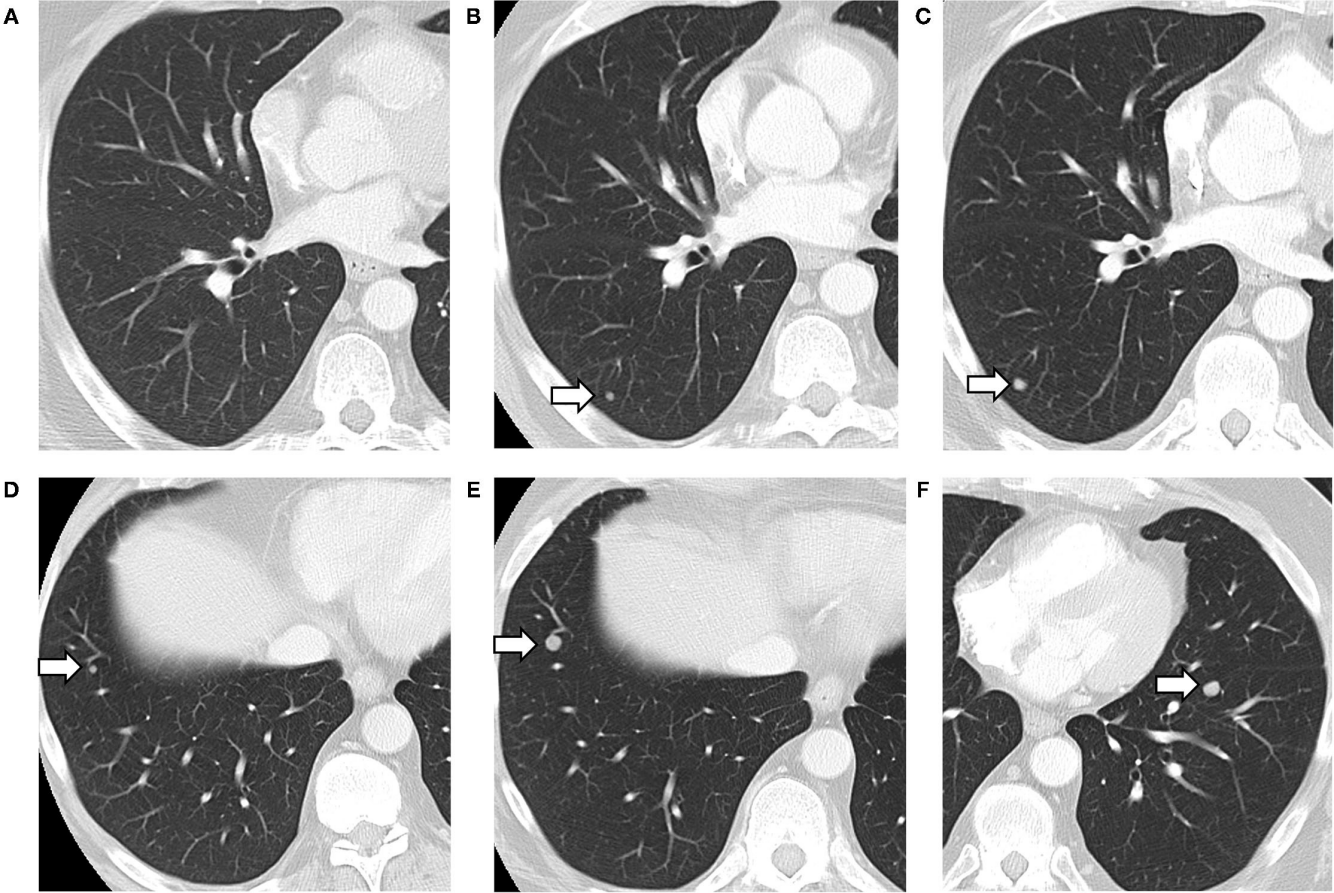
Lung metastases developing on serial CT scans (arrows). Axial images of the lung from three consecutive CT scans, showing the development of a lung nodule, in the posterior right lower lobe **(A–C)**. A separate nodule in the anterior right lower lobe also grew between the second **(D)** and third CT scan **(E)**. A third nodule appeared in the left lower lobe on the third scan only **(F)**. The first CT was negative for metastasis **(A)**, with the text in the “Lungs” section of the findings reading “No suspicious findings.” The model predicted correctly that there were no metastases described with 100% confidence. The second CT **(B,D)** was labeled as positive for metastases by the radiologist who had access to all three scans, but negative by the CNN (with a confidence of 99.60%) which only had reports for the first two. The third CT **(C,E,F)** was labeled as positive by both radiologists and the CNN (with 100% confidence).

Our study is not without limitations. It is important to note that the human annotators had access to slightly different information compared to what the models had access to at the time of prediction. The human annotators had access to both historical and future reports, while the models only had the text from previous reports concatenated to the target report to make their predictions. This resulted in false negative errors (example provided in Figure 3), though the model was able to correct the prediction for later reports. The implications of this depend on the use-case of the model. In the case where “future” reports (with respect to the target report) are available, such as in a retrospective study of disease pattern, exposing the model to these future reports would be desired. However, if the use-case is predicting the presence of metastases in a patient currently undergoing treatment and all reports to-date are presented to the model, the model is not missing any information.

In conclusion, the multi-report NLP prediction models presented in this paper generate more reliable weak labels of radiology reports compared with a single-report prediction model. The success of digital cancer twins relies heavily on the access of high-resolution representations of individual cancer patients over time. The ability to automatically generate accurate labels of metastatic disease from radiology reports will improve the viability of these digital twins, while enabling recognition of disease progression patterns through the availability of such a large database of generated weak labels. This will allow for earlier detection of potential progression of disease in individual patients allowing for more successful intervention during disease management.

## Data Availability Statement

The raw data supporting the conclusions of this article will be made available by the authors, without undue reservation.

## Author Contributions

KB, RD, LG, and AS: guarantors of integrity of entire study. RD, KB, KL, PC-A, FZ, and AS: literature research. RD and PC-A: clinical studies. KB, KL, LG, and AS: experimental studies. KB, KL, PC-A, LG, FZ, and AS: statistical analysis. All authors study concepts, study design or data acquisition or data analysis, interpretation, manuscript drafting or manuscript revision for important intellectual content, approval of final version of submitted manuscript, agrees to ensure any questions related to the work are appropriately resolved, and manuscript editing, and contributed to the article and approved the submitted version.

## Funding

Supported in part by National Institutes of Health/National Cancer Institute Cancer Center Support Grant P30 CA008748 and the New Frontiers in Research Fund Exploration Grant from Social Sciences and Humanities Research Council of Canada, and the Canada Research Chairs program.

## Conflict of Interest

LG activities not related to the present article; receives options for consultancy from Within Health. The remaining authors declare that the research was conducted in the absence of any commercial or financial relationships that could be construed as a potential conflict of interest.

## Publisher's Note

All claims expressed in this article are solely those of the authors and do not necessarily represent those of their affiliated organizations, or those of the publisher, the editors and the reviewers. Any product that may be evaluated in this article, or claim that may be made by its manufacturer, is not guaranteed or endorsed by the publisher.
